# Are Black and Latino adolescents being asked if they use electronic cigarettes and advised not to use them? Results from a community-based survey

**DOI:** 10.3389/fpubh.2023.1222184

**Published:** 2023-08-10

**Authors:** Margaret Connolly, Daniel Croft, Paula Ramírez-Palacios, Xueya Cai, Beverly Hill, Rafael H. Orfin, M. Patricia Rivera, Karen M. Wilson, Dongmei Li, Scott McIntosh, Deborah J. Ossip, Ana Paula Cupertino, Francisco Cartujano-Barrera

**Affiliations:** ^1^Department of Medicine, University of Rochester Medical Center, Rochester, NY, United States; ^2^Unidad de Investigación Epidemiológica y en Servicios de Salud Delegación Morelos, Instituto Mexicano del Seguro Social, Cuernavaca, Morelos, Mexico; ^3^Department of Biostatistics and Computational Biology, University of Rochester Medical Center, Rochester, NY, United States; ^4^Community Engagement Division, Ibero American Action League, Rochester, NY, United States; ^5^Department of Public Health Sciences, University of Rochester Medical Center, Rochester, NY, United States; ^6^Department of Pediatrics, University of Rochester Medical Center, Rochester, NY, United States; ^7^Department of Clinical and Translational Research, University of Rochester Medical Center, Rochester, NY, United States; ^8^Department of Surgery, University of Rochester Medical Center, Rochester, NY, United States

**Keywords:** vaping, electronic cigarettes, nicotine, tobacco, Black adolescents, Latino adolescents

## Abstract

**Objective:**

This study aimed to explore whether African American/Black and Hispanic/Latino adolescents are being asked about electronic cigarette (e-cigarette) use (vaping) and advised not to use them.

**Methods:**

In 2021, adolescents (*N* = 362) with no vaping history, self-identified as African American/Black and/or Hispanic/Latino, and able to read and speak English and/or Spanish were recruited through partner schools and community-based organizations. Participants completed a survey reporting sociodemographic characteristics (e.g., race/ethnicity, gender, and language of preference) and they were asked about e-cigarette use and/or were advised not to use them by a health professional.

**Results:**

In total, 12% of African American/Black and 5% of Hispanic/Latino participants reported not seeing a health professional in the year prior to enrollment. Of the participants who reported visiting a health professional, 50.8% reported being asked and advised about vaping. Over one-quarter (28.4%) of participants were neither asked nor advised regarding vaping. Compared to English-speaking participants, Spanish-speaking participants were significantly less likely to be asked about e-cigarette use (45.2 vs. 63.9%, *p* = 0.009) and advised not to use them (40.3 vs. 66.9%, *p* < 0.001). Moreover, compared to African American/Black participants, Hispanic/Latino participants were significantly less likely to be advised not to use e-cigarettes (52.9 vs. 68.6%, *p* = 0.018). Furthermore, compared to male participants, female participants were significantly less likely to be advised not to use e-cigarettes (51.3 vs. 68.2%, *p* = 0.003).

**Conclusion:**

Compared to English-speaking participants, Spanish-speaking participants were significantly less likely to self-report being asked about e-cigarette use and advised not to use them. Moreover, Hispanic/Latino and female adolescents were significantly less likely to self-report being advised not to use e-cigarettes compared to their Black/African American and male counterparts. Future research is needed to improve health professional attention toward asking about and advising against vaping among adolescents.

## 1. Introduction

Since 2014, electronic cigarettes (e-cigarettes) have been the most commonly used nicotine and tobacco product among middle and high school students in the United States (U.S.) (1). E-cigarette use (vaping) has been associated with adverse effects on brain development (2), heavy metal exposure (3), lung injury (4), and death (5). According to the 2022 U.S. National Youth Tobacco Survey, the prevalence of current (past 30-day) vaping among high school (grades 9–12) and middle school (grades 6–8) students was 14.1 and 3.3%, respectively (6, 7). Differences in the prevalence of current vaping exist among racial and ethnic groups (7). For example, among high school students, the prevalence of current vaping among White students (16.9%) is higher compared to African American/Black and Hispanic/Latino students (11.1 and 12.2%, respectively) (7). Interestingly, an opposite phenomenon exists among middle school students with African American/Black and Hispanic/Latino students having a higher prevalence of current vaping (4.1 and 4.2%, respectively) compared to White students (2.8%) (7).

Routine healthcare visits provide an opportunity to address vaping among adolescents. The Ask, Advise, and Refer (AAR) model is a well-established approach for health professionals worldwide to address nicotine and tobacco use among patients, including adolescents (8). The AAR model consists of the following steps in order: (1) ask all patients whether they use nicotine and tobacco, (2) advise patients who use nicotine and tobacco to quit, and (3) refer patients who use nicotine and tobacco to an evidence-based cessation program (9). If an adolescent does not use nicotine and tobacco, advising to prevent initiation of nicotine and tobacco use is recommended (10). The goal of this study was to explore whether African American/Black and Hispanic/Latino adolescents were being asked whether they use e-cigarettes and/or advised not to use them by health professionals. Moreover, this study examined the unique associations between being asked and/or advised about vaping and sociodemographic characteristics.

## 2. Methods

### 2.1. Study design

This study is a secondary data analysis of a randomized controlled trial (RCT) designed to assess the immediate impact of vaping prevention graphic messages on the susceptibility of future vaping among Black and Latino adolescents (11, 12). The RCT purposely focused on Black and Latino adolescents given their underrepresentation in previous vaping prevention studies (12). Details and results of the RCT have been reported elsewhere (12). The RCT was approved and monitored by the University of Rochester Medical Center Institutional Review Board (STUDY00006267).

### 2.2. Participants

In 2021, Black and Latino adolescents were recruited by a team of diverse, bilingual (English and Spanish), trained recruiters using proactive and reactive strategies. Adolescents aged 12–17 years with no history of e-cigarette use who self-identified as African American/Black and/or Hispanic/Latino and able to read and speak English and/or Spanish were included in the RCT. Parents'/guardians' permission and adolescents' assent were obtained from all subjects involved in the study. Details and results of the recruitment, eligibility, and permission/assent have been reported in a previous publication (13). Participants were compensated for their time with a $25 gift card.

### 2.3. Assessments

All assessments described in this study were completed in the language of preference of the participant, either English or Spanish, at baseline. The baseline survey collected information on demographics (e.g., age, gender, sexual preference, race, ethnicity, language of preference, grade, and parent or caregiver nicotine and tobacco use). The baseline survey also assessed whether participants were being asked if they use e-cigarettes and/or advised not to use them. Questions included “Think about when you have visited a doctor, dentist, nurse, or other health professional in the past 12 months. During any of these visits, were you asked if you use e-cigarettes?” and “Think about when you have visited a doctor, dentist, nurse, or other health professional in the past 12 months. Were you given advice not to use any e-cigarettes?” (14). Answer choices to both questions included “I did not see a doctor, dentist, nurse, or other health professional during the past 12 months,” “yes,” and “no.”

### 2.4. Analyses

For this study, participants who reported having not seen a health professional in the past 12 months were not included in the analyses. Sociodemographic characteristics of participants were described between those who were and were not asked about e-cigarette use and among those who were and were not advised about vaping. Fisher's exact test was used to compare proportions in categorical variables and crude odds ratios (OR) were reported. Student's *t*-test was used for continuous variables. Significant associations with being asked and/or advised about e-cigarette use (at the *p* < 0.05 level) were tested in an unadjusted logistic regression model, followed by a logistic regression model that adjusted for all significant associations. Analyses were conducted using Stata 15.0.

## 3. Results

At baseline (*N* = 362), 13.6% (*n* = 22) of African American/Black and 5.6% (*n* = 9) of Hispanic/Latino participants reported not having seen a health professional in the 12 months prior to enrollment. Of the participants who reported visiting a health professional in the past 12 months (*n* = 331), half (50.8%) reported both being asked and advised about e-cigarette use. An additional 20.9% of participants were either asked or advised. Over one-quarter of participants (28.4%) were neither asked nor advised regarding e-cigarette use.

Of the Hispanic/Latino participants who reported visiting a health professional in the previous 12 months, 64% of participants reported English as their preferred language and 36% reported Spanish as their preferred language. Hispanic/Latino participants who reported Spanish as their preferred language were significantly less likely to be asked (45.2 vs. 63.9%, *p* = 0.009) or advised (40.3 vs. 66.9%, *p* < 0.001) about e-cigarette use compared to primarily English-speaking Hispanic/Latino participants. Compared to African American/Black participants, Hispanic/Latino participants were less likely to be advised about e-cigarette use (52.9 vs. 68.6%, *p* = 0.018). Overall, female participants were less likely to be advised about e-cigarette use compared to male participants (51.3 vs. 68.2%, *p* = 0.003; Table 1).

**Table 1:** Sociodemographic characteristics of participants who were vs. who were not asked or advised about e-cigarette use.

	**Asked**	**Not asked**	***p*-value**	**Advised**	**Not advised**	***p*-value**
	***n*** = **200**	***n*** = **131**		***n*** = **205**	***n*** = **126**	
***Age** (Years, Mean)*	15.0	14.9	0.654	15.0	15.0	0.886
***Gender (n** **=** **328)***						
Male	62.6%	37.4%	0.291	68.2%	31.8%	**0.003**
Female	56.4%	43.6%		51.3%	48.7%	
***Sexual preference (n** **=** **313)***						
Heterosexual/straight	61.1%	38.9%	0.669	63.5%	36.5%	0.829
Non-heterosexual/non-straight	68.0%	32.0%		60.0%	40.0%	
***Race/ethnicity***						
African American/Black	62.3%	37.7%	0.574	68.6%	31.4%	**0.018**
Hispanic/Latino	58.7%	41.3%		52.9%	44.2%	
***Language of preference***						
English	63.9%	36.1%	**0.009**	66.9%	33.1%	**< 0.001**
Spanish	45.2%	54.8%		40.3%	59.7%	
***Grade***						
6th−9th	56.6%	43.4%	0.295	62.3%	37.7%	1.000
10th−12th	62.7%	37.3%		61.7%	38.3%	
***Parent or caregiver nicotine and tobacco use***						
At least one parent/caregiver currently smokes tobacco-related products	60.0%	40.0%	1.0	56.0%	44.0%	0.348
At least one parent/caregiver currently uses e-cigarettes	72.7%	27.3%	0.138	72.7%	27.3%	0.192
No parent/caregiver currently smokes tobacco-related products or e-cigarettes	59.4%	40.6%	0.595	62.6%	37.4%	0.689

Bold indicates statistical significance.

Differences persisted after adjusting for significant associations with being asked and/or advised about e-cigarette use (i.e., gender, race/ethnicity, and language of preference), with Spanish-speaking adolescents self-reported as being significantly less likely to be asked (AOR = 0.43, 95% CI: 0.22–0.81) and advised (AOR = 0.39, 95% CI: 0.20–0.75) about e-cigarette use in comparison to English-speaking adolescents. Similarly, female participants remained significantly less likely to be advised about e-cigarette use by a health professional when compared to male participants (AOR = 0.52, 95% CI: 0.32–0.85). Finally, Hispanic/Latino adolescents were significantly less likely to be advised relative to African American/Black adolescents (OR = 0.58, 95% CI: 0.37–0.91; Table 2).

**Table 2:** Impact of sociodemographic factors on adolescents' self-reported rates of being asked or advised by a health professional about e-cigarette use.

	**Asked**		**Advised**	
	**Model 1 unadjusted (OR, 95% CI)**	**Model 2 adjusted (AOR, 95% CI)** ^a^	**Model 1 unadjusted (OR, 95% CI)**	**Model 2 adjusted (AOR, 95% CI)** ^a^
**Gender**				
Male	Reference	Reference	Reference	Reference
Female	0.77 (0.49–1.23)	0.85 (0.53–1.37)	**0.49 (0.31–0.78)** ^**^	**0.52 (0.32–0.85)** ^**^
**Race/ethnicity**				
African American/Black	Reference	Reference	Reference	Reference
Hispanic/Latino	0.86 (0.55–1.34)	1.23 (0.73–2.08)	**0.58 (0.37–0.91)** ^*^	0.82 (0.48–1.40)
**Language of preference**				
English	Reference	Reference	Reference	Reference
Spanish	**0.46 (0.27–0.81)** ^**^	**0.43 (0.22–0.81)** ^*^	**0.33 (0.19–0.59)** ^**^	**0.39 (0.20–0.75)** ^**^

OR, odds ratio; 95% CI, 95% confidence interval; AOR, adjusted odd ratio. “a” indicates that Model 2 controls for the variables gender, race/ethnicity, and language of preference. ^*^p < 0.05. ^**^ p < 0.01. Bold indicates statistical significance.

## 4. Discussion

To the best of our knowledge, this is the first study to assess (1) whether African American/Black and Hispanic/Latino adolescents are being asked whether they use e-cigarettes and/or advised not to use them and (2) the unique associations of being asked and/or advised about vaping with sociodemographic characteristics. In this study, only half of the adolescents who reported having seen a health professional within the previous year self-reported being both asked and advised about e-cigarette use. Furthermore, one-quarter of the participants were neither asked nor advised regarding e-cigarette use. Primarily Spanish-speaking adolescents self-reported significantly lower rates of being asked and advised about e-cigarette use during healthcare visits when compared to English-speaking adolescents. This difference persisted even after controlling for significant associations with being asked and/or advised about e-cigarette use. Additionally, Hispanic/Latino and female participants self-reported lower rates of being advised about e-cigarette use in comparison to African American/Black and male participants, respectively.

There are many possible factors contributing to this low rate of addressing e-cigarette use at healthcare visits. Electronic health record tools have the potential to assist with reminding health professionals to address smoking and vaping among adolescents. However, the use of these tools remains low, with a 2019 study finding that, on review of 518 adolescent well visits, none documented assessment of e-cigarette use (15). Moreover, the content and use of electronic health record tools are inconsistent. Additionally, health professionals often report discomfort addressing e-cigarette use at healthcare visits (16). While 11% of health professionals reported providing care for an adolescent who had used e-cigarettes, most reported moderately low comfort levels discussing e-cigarette use with adolescents (16). In a second study evaluating primary care professionals, only 58% of them reported feeling comfortable addressing e-cigarette use with adolescent patients (17). These primary care professionals reported that additional training was necessary to clarify how best to address vaping and to review chemicals present in e-liquids (17).

Our study showed that female participants did not significantly differ from male participants in the likelihood of being asked about e-cigarette use. However, female participants were significantly less likely to be advised against e-cigarette use when compared to male participants. One possible reason for this observed difference is that health professionals may perceive that vaping is higher among male adolescents compared to their female counterparts. However, according to the 2022 U.S. National Youth Tobacco Survey, the prevalence of current vaping is higher among female adolescents compared to male adolescents (10.5 vs. 8.3%, respectively) (7). Additionally, e-cigarette distributors have implemented marketing strategies such as promoting the use of e-cigarettes for weight loss and producing slim and pink devices targeting female consumers (18). Addressing e-cigarette use among female adolescents at healthcare visits presents an important opportunity to reduce current vaping.

Spanish-speaking adolescents in this study were significantly less likely to be asked or advised about e-cigarette use in comparison to English-speaking adolescents. Language barriers and poor access to or quality of translation services have previously been identified as significant factors contributing to reduced smoking cessation support provided to Spanish-speaking Latino patients (19). These factors likely contribute similarly to disparities in addressing e-cigarette use among primarily Spanish-speaking adolescents.

There are several limitations to this study. First, data are self-reported, and there is a possibility that participants felt compelled to offer socially desirable responses or did not remember whether they were asked and/or advised about vaping. Second, the study did not assess who asked and/or advised the adolescent about vaping (e.g., the doctor, dentist, nurse, and/or other health professional). This information could guide future-targeted interventions to increase the AAR model. Third, given that this study focused on adolescents who have not vaped, we did not assess whether participants were referred—the third pillar of the AAR model—to vaping cessation resources. Fourth, the exclusion of adolescents who did not self-identify as African American/Black and/or Hispanic/Latino (e.g., non-Hispanic/Latino white adolescents) prevented the study from shining light on other potential racial and ethnic disparities. Lastly, this is a non-probability sample of African American/Black and Hispanic/Latino adolescents which limits generalizability to the broader population of Black and Latino adolescents.

## 5. Conclusion

In this study, compared to English-speaking participants, Spanish-speaking participants were significantly less likely to self-report being asked about e-cigarette use and advised not to use them. Moreover, Hispanic/Latino and female adolescents were significantly less likely to self-report being advised not to use e-cigarettes compared to their Black/African American and male counterparts. Future research is needed to improve health professionals' attention to asking about and advising against vaping among adolescents.

## Data availability statement

The datasets generated for this study are available on request to the corresponding author.

## Ethics statement

The studies involving human participants were reviewed and approved by University of Rochester Medical Center. Written informed consent for participation was not provided by the participants' legal guardians/next of kin because: Parents'/guardians' permission and adolescents' assent were obtained from all subjects involved in the study.

## Author contributions

MC, DC, SM, DO, AC, and FC-B: conceptualization. PR-P and XC: formal analysis. RO: project administration. MC, DC, and FC-B: writing—original draft preparation. PR-P, XC, BH, RO, MR, KW, DL, SM, DO, and AC: writing, reviewing, and editing. All authors have read and agreed to the published version of the manuscript.
